# Willingness to participate in vaccine trials among MSM in São Paulo, Brazil

**DOI:** 10.1186/1742-4690-9-S2-P231

**Published:** 2012-09-13

**Authors:** G Calazans, R Gamboa, A Kalichman, M Ribeiro, M Veras

**Affiliations:** 1 Sao Paulo HVTU - CRT-DST/AIDS, São Paulo, Brazil; 2 School of Medical Sciences, Santa Casa de São Paulo, Brazil

## Background

An effective HIV vaccine still poses a great challenge to research scientists. Among the most vulnerable populations to HIV infection in Europe, United States and Latin America are men who have sex with men (MSM), potential volunteers for HIV vaccine research. We have asked MSM from a large LA city about their willingness to participate (WTP) in HIV vaccine trials.

## Methods

A survey of men who attend MSM-identified venues in downtown São Paulo was carried out between November 2011 and January 2012. Time-location sampling approach was used. During sampling events, interviewers approached and asked men to respond a brief eligibility interview and consecutively applied a questionnaire to men who referred having ever anal or oral sex with men or transvestite. Here we analyze WTP in hypothetical HIV preventative vaccine trials.

## Results

1217 MSM were interviewed (missing data=3), 54,6% would accept participating, 35,3% would deny, and 9,9% stated they were unsure/maybe/depending on more information or assurances before deciding whether to participate in vaccine trials. WTP in HIV vaccine trial was similar across age groups (p=.450) and race/ethnicity (p=.534). There was no patterned difference among those who self-identified as homosexual, bisexual or transgender (p=.542) as well as among those who would have sex with an HIV-infected person, the size of their HIV-infected network (p=.251). Although not significant (p=0.06) a trend was suggested with WTP and the level of education of the head of the household, as years of education increased greater WTP.

## Conclusion

Findings indicate that, despite the lack of success in recent phase II/III trials, there is substantial WTP in HIV vaccine studies, expressing the hope for an effective vaccine retain a pool of potential volunteers among the most vulnerable population. Subsequent analysis should focus on association with behavioral aspects, as risk behaviors, experience of discrimination due to sexuality and WTP.

